# The Effect of Opioids and Benzodiazepines on Exacerbation Rate and Overall Survival in Patients with Chronic Obstructive Pulmonary Disease on Long-Term Non-Invasive Ventilation †

**DOI:** 10.3390/jcm13185624

**Published:** 2024-09-22

**Authors:** Andrew Chai, Balazs Csoma, Zsofia Lazar, Andrew Bentley, Andras Bikov

**Affiliations:** 1 Division of Immunology, Immunity to Infection and Respiratory Medicine, University of Manchester, Manchester M13 9PL, UK; andrew.chai2@nhs.net (A.C.); andrew.bentley@mft.nhs.uk (A.B.); 2 Wythenshawe Hospital, Manchester University NHS Foundation Trust, Manchester M23 9LT, UK; csoma.balazs@semmelweis.hu; 3 Department of Pulmonology, Semmelweis University, 1083 Budapest, Hungary; lazar.zsofia@med.semmelweis-univ.hu

**Keywords:** COPD, benzodiazepines, opioids, respiratory failure, non-invasive ventilation

## Abstract

**Background**: There is a growing concern that opioids and benzodiazepines can depress the respiratory drive and could contribute to worsening respiratory failure and higher exacerbation frequency in COPD. However, the relationship between the exacerbation rate and medication taken is poorly understood in patients with chronic respiratory failure due to COPD. **Methods**: As part of a service evaluation project, we analysed 339 patients with COPD who were established on long-term non-invasive ventilation (LT-NIV) at our tertiary centre. We investigated the relationship between benzodiazepine and opioid prescription and clinical outcomes as well as their impact on the exacerbation rate and overall survival following setup. **Results**: Before LT-NIV setup, 40 patients took benzodiazepines and 99 patients took opioids. Neither benzodiazepine nor opioid use was associated with changes in daytime blood gases, overnight hypoxia or annual exacerbations before NIV setup, but patients taking opioids were more breathless as assessed by modified Medical Research Council scores (3.91 ± 0.38 vs. 3.65 ± 0.73, *p* < 0.01). Long-term NIV significantly reduced the number of yearly exacerbations (from 3.0/2.0–5.0/ to 2.8/0.71–4.57/, *p* < 0.01) in the whole cohort, but the effect was limited in those who took benzodiazepines (from 3.0/2.0–7.0/ to 3.5/1.2–5.5/) or opioids (3.0/2.0–6.0/ to 3.0/0.8–5.5/). Benzodiazepine use was associated with reduced exacerbation-free survival and overall survival (both *p* < 0.05). However, after adjustment with relevant covariates, the relationship with exacerbation-free survival became insignificant (*p* = 0.12). Opioids were not associated with adverse outcomes. **Conclusions**: Benzodiazepines and opiates are commonly taken in this cohort. Whilst they do not seem to contribute to impaired gas exchange pre-setup, they, especially benzodiazepines, may limit the benefits of LT-NIV.

## 1. Introduction

Chronic obstructive pulmonary disease (COPD) is a common, usually progressive disease of the airways and lung parenchyma that is characterised by non-reversible airflow limitation. It affects around 10% of the adult population worldwide [1] and is considered the third leading cause of death [2]. Breathlessness is a common symptom for COPD, which is a result of a multifactorial process, with various physiologies culminating in a discrepancy between the inspiratory drive and mechanical ventilatory response [3,4]. Dyspnoea is strongly related to disease severity [5] and is also associated with the risk of exacerbation and mortality [6,7]. It is a debilitating symptom which affects nearly all patients with end-stage disease [8].

Patients with COPD may experience acute deteriorations, termed COPD exacerbations, which also correlate with disease severity [9]. Whilst these events are usually considered inflammatory, in 10% of cases they occur without changes in markers of inflammation [10,11]. Pauci-inflammatory COPD exacerbations are less well investigated, and it is possible that undetected inflammatory pathways play a role in these. However, in some cases, they may involve non-inflammatory factors related to impaired respiratory mechanics, reduced respiratory drive and impaired mucociliary clearance.

Whilst opioids effectively improve breathlessness [12], there is no convincing evidence that benzodiazepines are effective in relieving dyspnoea in COPD, despite their common use for this indication [13]. In addition, benzodiazepines are used to treat anxiety and insomnia, and opioids are prescribed for chronic pain, problems which often co-exist with COPD [14]. However, these medications may depress the respiratory drive and may contribute to respiratory failure [15,16]. In addition, opioids may suppress the cough reflex [17] and benzodiazepines may reduce the ciliary beat frequency [18], contributing to impaired clearance of mucus. Both medications are associated with increased exacerbation rate and mortality in COPD [19,20,21]. As a result, these drugs are also often underutilised, even in patients with end-stage disease [22].

With disease progression, patients with COPD are susceptible to developing hypercapnia [23]. Hypercapnia in patients with COPD is associated with increased mortality [20] and increased susceptibility to chest infections [23]. In line with this, normalising hypercapnia in COPD using long-term non-invasive ventilation (LT-NIV) improves survival [24,25,26] and reduces the exacerbation rate [24]. It is unclear, however, how opioids and benzodiazepines affect the effectiveness of LT-NIV in patients with COPD, as patients taking these medications are either excluded in randomised controlled trials, medications are not reported, or their effects are not analysed [24,25,26]. Whilst benzodiazepines could worsen respiratory failure and contribute to adverse events, effective ventilation may counteract this effect, potentially mitigating their risk. To the best of our knowledge, only one retrospective study has investigated these medications in patients with COPD on LT-NIV, which showed that opioid and benzodiazepine use was associated with decreased survival. However, this study did not analyse these medications separately, and neither were exacerbations studied [27]. 

The aim of this service evaluation project was to understand the prevalence of benzodiazepine and opioid use in patients with COPD set up on LT-NIV, as well as to analyse the impact of these medications on overall survival and exacerbation-free survival post-LT-NIV setup in order to improve the quality of care of patients under our service.

## 2. Materials and Methods

### 2.1. Project Design and Subjects

**Figure 1:**
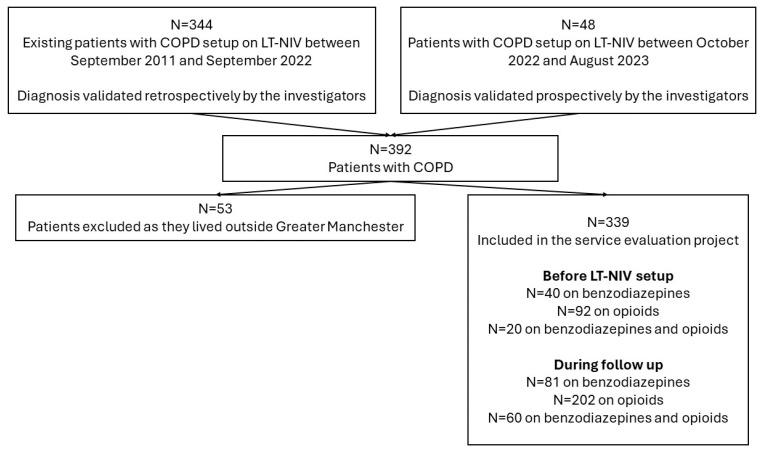
Patient inclusion chart.

We assessed the data of 392 patients with COPD who were set up on long-term non-invasive ventilation at the North West Ventilation Unit, Wythenshawe Hospital, Manchester University NHS Foundation Trust, Manchester, United Kingdom, a regional tertiary service. The group consisted of historical patients who were alive in September 2022 (set up on LT-NIV between September 2011 and September 2022, n = 344 at our service) and 48 patients who were set up on LT-NIV between October 2022 and August 2023 and were prospectively followed up until April 2024. Clinical data, including comorbidities and medication lists, were obtained from the in-hospital electronic health records and the Greater Manchester Care Records. As 53 patients lived outside Greater Manchester, we excluded these patients from this analysis to avoid inaccuracies in medications and unreported exacerbations. As a result, the data of 339 patients were analysed. For patient selection see Figure 1. 

COPD was diagnosed historically based on symptoms, suggestive medical history and lung function. However, lung function results, including forced expiratory volume in 1 s (FEV_1_) and forced vital capacity (FVC), were available only in 220 patients. The diagnosis was validated by the clinical team during their admission for LT-NIV setup and as part of this service evaluation project by the investigators. Comorbidities were defined based on electronic health records and patient medications. Based on these, we computed the Charlson comorbidity index (CCI) [28]. We assessed the number of moderate-to-severe exacerbations from the medical records in the year before LT-NIV setup as well as post-setup. The exacerbations were defined by either systemic corticosteroid and/or antibiotic use. If patients required repeated courses of antibiotics or corticosteroids within 30 days, this was considered a single event. Exacerbations were cross-matched with patient reports during their scheduled follow-ups (every 3, 6 or 12 months, depending on the clinical need). Data on hospitalisations to respiratory wards due to exacerbations was available from Greater Manchester Health records and they were cross-matched with the patients’ reports. Before LT-NIV setup, we assessed their symptoms based on the modified Medical Research Council (mMRC) questionnaire and their frailty based on the Clinical Frailty Score (CFS). Two-hundred and fifty patients had nocturnal oximetry before setup, we recorded the 4% oxygen desaturation index (ODI) and the percentage of time spent with oxygen saturation below 90% (T90%). Blood gases were determined in arterialised capillary blood gas samples.

Following assessment using the Medical Research Council decision tool (https://www.hra-decisiontools.org.uk/research/), the project was registered as a service evaluation (HL075) at Wythenshawe Hospital, Manchester University Foundation Trust on 16 October 2022. Data collection and analysis adhered to the Caldicott Principles. Service Evaluation projects when conducted are part of the Trust’s Public Task in-order-to inform evidence-based practice and improve patient care, relying on Articles 6.1(e) and 9.2(h). The Trust does not rely on explicit consent when conducting service evaluation studies.

This article is a revised and expanded version of a conference abstract entitled “Benzodiazepines and Opiates Shorten Exacerbation-Free Time in COPD Patients on Long-Term Non-Invasive Ventilation”, which was presented at the SLEEP 2024 conference, Houston, 1–5 June 2024 [29].

### 2.2. Statistical Analysis

JASP 0.14 (JASP Team, University of Amsterdam, Amsterdam, the Netherlands) and Statistica 12 (StatSoft, Inc., Tulsa, OK, USA) software were used for statistical analysis. Demographics and clinical characteristics were compared with Mann–Whitney and Chi-square tests. The change in the number of annualised exacerbations was analysed with the Wilcoxon test. Kaplan–Meier curves were computed to investigate exacerbation-free survival and overall survival. The Gehan’s test was used to compare survival curves, whilst univariate and multivariate Cox-regression analyses identified factors associated with exacerbation-free survival and overall survival. A priori power or sample size calculations were not performed, as this was a service evaluation project of the routine clinical practice. Data are expressed as mean ± standard deviation and median/interquartile range/; *p* < 0.05 was considered significant.

## 3. Results

### 3.1. Baseline Characteristics

The cohort consisted of predominantly middle-aged women who had severe to very severe COPD, who were highly symptomatic with a high number of exacerbations, borderline daytime and severe nocturnal hypoxia and chronic daytime hypercapnia (Table 1). Before the long-term NIV setup, 40 patients used benzodiazepines (27 lorazepam, 15 diazepam, four temazepam, three nitrazepam, two clonazepam and one clobazam, with 12 patients using two benzodiazepines at the same time) and 92 using opioids (40 morphine sulphate, 30 codeine, 20 methadone, 12 buprenorphine, 12 tramadol, eight oxycodone and one oxybutinin, with 31 using two opioids). Twenty patients used both groups of medications. Patients who took benzodiazepines were leaner and had a higher prevalence of anxiety, but interestingly had lower CCI and ODI. Patients who used opioids were younger, had a higher prevalence of anxiety and had higher breathlessness scores on the mMRC, but their clinical characteristics were similar to those who did not use opioids. Patients on benzodiazepines who also took opioids had similar characteristics, but they also had more severe overnight hypoxia compared to those patients who only took benzodiazepines without opioids. Patients on opioids who also took benzodiazepines were leaner but had similar characteristics. Most notably, compared to benzodiazepines or opioids alone, anxiety was significantly more prevalent in the group with combined drug use. 

### 3.2. Follow-Up Data

During their follow-up, a further 41 patients were started on benzodiazepines and 110 patients on opioids; by the end of the follow-up period, 60 patients took both groups of medications. 

The number of annualised exacerbations significantly decreased following LT–NIV setup in the whole cohort (from 3.0/2.0–5.0/to 2.8/0.71–4.57/, *p* < 0.01). However, those patients who took benzodiazepines (in total 81 patients, 0.50 mg/0.25–0.75/mg clonazepam equivalent) or opioids (in total 202 patients, 24 mg/12–44/mg morphine equivalent) did not experience this benefit (from 3.0/2.0–7.0/to 3.5/1.2–5.5/and from 3.0/2.0–6.0/to 3.0/0.8–5.5/, benzodiazepines and opioids, respectively). In contrast, in patients who were not using either benzodiazepines or opioids (n = 116), the number of exacerbations decreased significantly (from 3/2–5/to 2/0–4/, *p* = 0.02). 

### 3.3. Exacerbation-Free Survival

**Figure 2:**
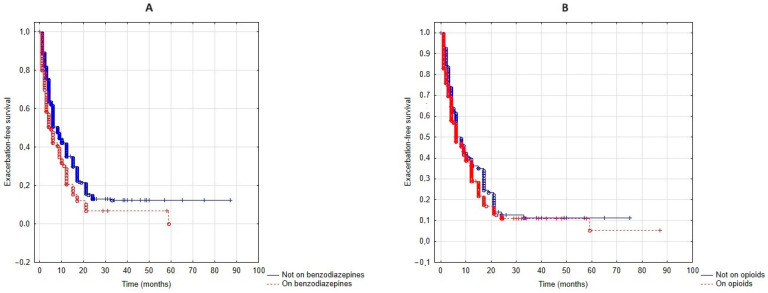
Exacerbation-free survival when patients on and off benzodiazepines (**A**) as well as on and off opioids (**B**) were compared.

Patients who took benzodiazepines experienced shorter exacerbation-free survival (*p* < 0.01, Figure 2A). In contrast, patients who took opioids had similar exacerbation-free survival (*p* = 0.14, Figure 2B). 

The Cox regression analyses revealed that lower body mass index (BMI, β = −0.02, *p* < 0.01), the number of exacerbations in the year pre–NIV setup (β = 0.09, *p* < 0.01), mMRC (β = 0.37, *p* < 0.01), CFS (β = 0.19, *p* = 0.01), FEV_1_ (β = −0.01, *p* = 0.02) and benzodiazepines (β = 0.36, *p* = 0.02) were significantly related to exacerbation–free survival, while a higher ODI tended to be protective (β = −0.01, *p* = 0.07). There was no relationship with age, sex, CCI, pre–setup blood gases, opioid use and T90%. When the Cox–regression model on benzodiazepines and exacerbation-free survival was adjusted for BMI, the number of exacerbations in the year pre–NIV setup, mMRC, CFS, FEV_1_ and ODI, the association became insignificant (*p* = 0.12). In this multivariate model, only the number of exacerbations in the year pre–NIV setup was a significant predictor (β = 0.07, *p* < 0.01).

### 3.4. Overall Survival

**Figure 3:**
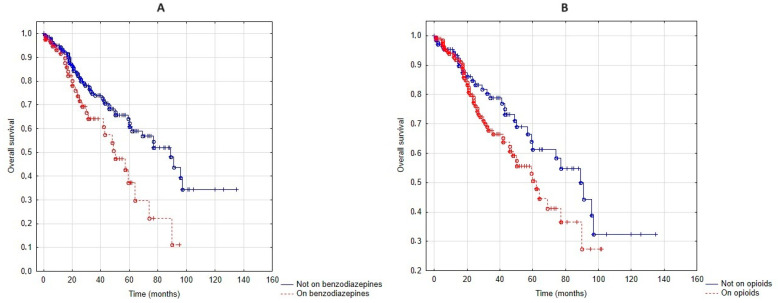
Overall survival when patients on and off benzodiazepines (**A**) as well as on and off opioids (**B**) were compared.

Patients who took benzodiazepines trended towards shorter survival (*p* = 0.08, Figure 3A), while there was no significant difference in those who took or did not take opioids (*p* = 0.26, Figure 3B).

Using Cox regression, the overall survival was significantly associated with age (β = 0.03, *p* = 0.02), BMI (β = −0.06, *p* < 0.01), the number of exacerbations in the year pre–NIV setup (β = 0.13, *p* < 0.01), mMRC (β = 0.86, *p* = 0.02), CFS (β = 0.84, *p* < 0.01), ODI (β = −0.07, *p* < 0.01) and benzodiazepines (β = 1.20, *p* < 0.01), while there was a trend for an association between higher CCI (β = 0.13, *p* = 0.07), and lower FEV_1_ (β = −0.02, *p* = 0.08). There was no association with sex, pre–setup blood gases and opioid dose. When the Cox regression model for benzodiazepines and overall survival was adjusted for age, BMI, the number of exacerbations in the year pre–NIV setup, mMRC, CCI, CFS, FEV_1_ and ODI, the association was still significant (β = 0.97, *p* < 0.01). In this multivariate model, BMI (β = −0.04, *p* < 0.01), CFS (β = 0.60, *p* < 0.01) and ODI (β = −0.04, *p* = 0.04) were also independent predictors.

### 3.5. The Effect of Combined Benzodiazepine and Opioid Use on Exacerbation-Free Survival and Overall Survival

**Figure 4:**
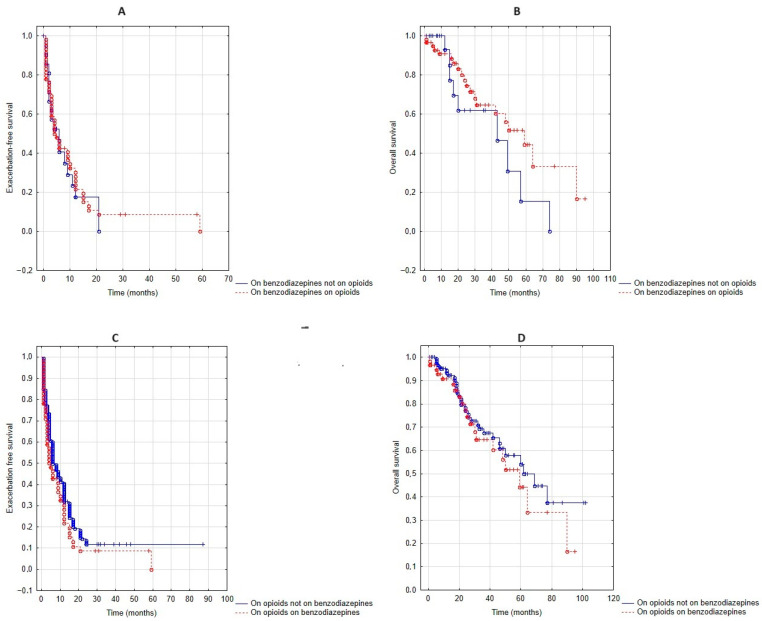
Combined use of benzodiazepines and opioids. Exacerbation-free survival when patients on benzodiazepines, but on and off opioids (**A**), as well as patients on opioids, but on and off benzodiazepines (**C**) were compared. Overall survival when patients on benzodiazepines, but on and off opioids (**B**), as well as patients on opioids, but on and off benzodiazepines (**D**) were compared.

Analysing the 81 patients who took benzodiazepines, the additional use of opioids did not affect exacerbation-free survival (*p* = 0.97, Figure 4A) or overall survival (*p* = 0.68, Figure 4B). In contrast, in those 202 patients who took opioids, the addition of benzodiazepines tended to decrease exacerbation-free survival (*p* = 0.09, Figure 4C), but did not significantly affect overall survival (*p* = 0.44, Figure 4D).

## 4. Discussion

Our service evaluation project investigated the prevalence of opioid and benzodiazepine prescriptions and their effect on exacerbations and mortality in an attempt to improve the safety of prescribing these medications when treating patients with COPD on LT–NIV. We found that, despite the concerns related to respiratory drive depression, these drugs are commonly prescribed in patients with chronic hypercapnic respiratory failure. As expected, patients on opioids complained about more significant dyspnoea and had a higher prevalence of anxiety, and benzodiazepines were most often prescribed for anxiety. Interestingly, in contrast to a former large study [30], their usage was not associated with worse COPD outcomes, such as the exacerbation rate or the severity of respiratory failure, apart from overnight hypoxia. However, as nocturnal hypoxia may be associated with worse outcomes for symptoms and overall health [31], further analyses should explore the impact of benzodiazepines and opioids on nocturnal respiratory function and assess the role of long-term non-invasive ventilation therapy in mitigating these effects. 

Opioids are commonly taken by patients with COPD at the advanced disease stage [32]. This was replicated by our project. There is a significant concern that these medications may lead to drug overdose, respiratory depression, hospitalisation and death in COPD [19]. However, the effect is dose-dependent, and lower-dose opioids (≤30 mg) were not associated with increased mortality in patients with COPD [30]. In the current service evaluation project, we did not find any association between opioid prescription and signs of respiratory depression such as daytime or night-time hypoxia or daytime hypercapnia, neither was there a significant relationship between opioid use and post-NIV exacerbation rates or overall survival. The latter could suggest that LT–NIV can effectively mitigate the respiratory drive depression associated with opioids and that, with appropriate caution, opioids can safely be prescribed for patients with COPD who are established on long–term NIV. Of note, the median dose of opioids was 24 mg morphine equivalent, and it is possible that higher doses would be associated with adverse outcomes. Whilst opioids did not significantly affect overall survival, this association could have been underpowered in this service evaluation project. Due to the limited number of subjects, we did not investigate individual opioids, and it is possible that some may be more harmful than the others.

In contrast, patients taking benzodiazepines experienced shorter time to the next exacerbation and reduced survival following LT–NIV setup. The exacerbation-free survival [21,33] and the overall survival [30] data are in line with previous reports in a general COPD cohort and in patients on LT–NIV [27]. It is important to note that while the relationship between benzodiazepine use and mortality strengthened following adjustment, the association with exacerbations disappeared. In the multivariate model, previous exacerbations predicted exacerbation-free survival in line with previous studies [34,35], emphasising the need to optimise pharmacological and non–pharmacological treatment to reduce the re–exacerbation rate. Nevertheless, the potential relationship between benzodiazepines with exacerbation, supported by previous studies [21,33], needs to be considered with caution. Firstly, benzodiazepines may cause hypoventilation—contributing to alveolar collapse—and can consequently worsen hypercapnia [16], which can paralyse the immune system, contributing to infections [23]. Secondly, benzodiazepines prolong apnoeas [36], which can provoke micro–aspiration and subsequent chest infections [37]. Thirdly, benzodiazepines can impair mucociliary clearance [18,38], which can result in both mechanical obstruction and infections. Whilst LT–NIV can effectively mitigate hypercapnia and apnoeas, its effect is limited in secretion management. Unfortunately, our project neither investigated the potential causes of exacerbations, nor did it phenotype the acute events. In addition, specific benzodiazepines were not investigated in this project separately. 

Benzodiazepines are commonly used together with opioids, and their combined use is a significant concern [39]. A previous, large–cohort study on Medicare beneficiaries concluded that compared to opioids alone, the combination of these drugs was associated with adverse respiratory events [40]. In our cohort, six percent were using this combination already before LT–NIV setup, and the prevalence increased to 18% during the follow-up. Whilst the combination was not associated with worse respiratory failure at daytime, it resulted in worse overnight hypoxia when compared to single medications. Notably, this adverse effect can effectively be mitigated with LT–NIV. During the follow-up, while adding an opioid to benzodiazepines did not affect exacerbations and overall survival, introducing benzodiazepines to opioids tended to worsen exacerbation-free survival. Although the effect was not significant, it raises concerns, especially that based on animal experiments, benzodiazepines and opioids can have a synergistic effect on respiratory drive depression [41]. Nevertheless, our findings were in line with previous results on the combined use of opioids with benzodiazepines, namely that this combination does not elevate the mortality risk further [30]. 

Our results replicated some results of former studies investigating factors associated with mortality in patients with COPD on LT–NIV [27,42]. Most importantly, a higher BMI was protective for mortality. This, together with the inverse relationship between ODI and mortality, suggests that some positive effects of LT–NIV are accounted for by the treatment of concomitant obstructive sleep apnoea [43], a disease that was excluded from randomised controlled trials on LT–NIV in COPD [24,25,26]. This was further clarified when a multivariate Cox–regression was applied, identifying BMI and ODI as independent preventive factors. Further parameters, such as the exacerbation rate, symptoms and airflow obstruction, are well-known factors associated with mortality in COPD [44,45]. 

Although we did not record the indication for drug prescription, we noted that breathlessness and anxiety were associated with their use. Unfortunately, we did not evaluate alternative reasons for opioid and benzodiazepine use, such as chronic pain or insomnia, that can bias the association between dyspnoea and opioid as well as benzodiazepine use. In addition, the large number of patients on methadone suggests that these patients were previous heroin users. Nevertheless, all patients complained about a significant degree of breathlessness that was both associated with shorter time to exacerbation and death. It is possible that frail patients in our cohort had very limited reserves, and even a minimal worsening of their breathlessness was perceived as an exacerbation [6]. Our results have also replicated previous findings showing that dyspnoea is significantly associated with survival in COPD [7].

This was a single centre service evaluation project that did not intend to produce generalisable results. Some of the analyses could have been underpowered due to the low number of events; therefore, further caution is warranted when prescribing opioids and benzodiazepines. In addition, lung function and overnight oximetry data was missing in some individuals. Clinical parameters were taken before LT–NIV setup, and we did not assess their temporal changes. Furthermore, benzodiazepines and opioids were introduced at different time points during the follow-up, and doses were up-titrated in some patients. The statistical analyses did not take into account these variations. We relied on an external database (Greater Manchester Care Records) when evaluating exacerbations as well as opioid and benzodiazepine use. We cross–matched these results with patients’ reports to minimise the bias. However, it is possible that some exacerbations were missed. More importantly, the database relied on prescriptions given by general practitioners (GPs). Opioids and benzodiazepines are often initiated directly by specialist teams (i.e., respiratory medicine, palliative care, mental health, etc.) and the prescription is later taken over by the GPs. Conversely, it is possible that the GPs prescribed these medications for future use (as necessary), and patients started to take them later. As a result, we did not include data on when these medications were initiated during the follow-up. Furthermore, the project was limited in its sample size and geographical area (Greater Manchester), and an adjustment with covariates in a larger cohort may reveal the relationship between medication use and outcomes more precisely. Therefore, the findings need to be confirmed in multi-centre randomised controlled trials.

Our service evaluation project has confirmed the high prevalence of benzodiazepine and opioid usage in patients with COPD using LT–NIV. The project has also revealed safety concerns, especially with benzodiazepine use. On one hand, our results should prompt more strict prescription rules for prescribing medications that can depress respiratory drive in patients with chronic hypercapnic respiratory failure. On the other hand, post-LT–NIV data on exacerbation and mortality should be analysed in conjunction with opioid and benzodiazepine use. We did not explore the underlying mechanisms in detail. Future research involving animal or cellular models could further investigate the effects of these medications on respiratory function and the respiratory centre in patients with COPD. This could offer deeper insights into how these drugs influence clinical outcomes.

## Figures and Tables

**Table 1:** Patient characteristics.

	Patients on Benzodiazepines (n = 40)	Patients Not on Benzodiazepines (n = 299)	*p*-Value 1	Patients on Opioids (n = 92)	Patients Not on Opioids (n = 247)	*p*-Value 2	Patients on Both Benzodiazepines and Opioids (n = 20)	*p*-Value 3	*p*-Value 4
Age (years)	65/60–71/	65/59–71/	0.97	62/56–70/	66/60–72/	<0.01	67/62–71/	0.89	0.17
Sex (% females)	63	58	0.58	59	58	0.95	65	0.74	0.52
BMI (kg/m^2^)	27.8/22.0–33.8/	32.8/25.1–39.7/	<0.01	30.2/23.7–41.3/	32.7/24.9–38.1/	0.79	26.7/23.9–30.0/	0.82	0.04
Number of exacerbations in the year pre–NIV setup	3.5/2.8–7.0/	3.0/2.0–5.0/	0.16	3.0/2.0–7.0/	3.0/2.0–7.0/	0.23	4.0/3.0–7.0/	0.25	0.34
CCI	1.0/1.0–2.0/	2.0/1.0–3.0/	0.04	2.0/1.0–2.0/	2.0/1.0–3.0/	0.10	1.0/1.0–2.0/	0.26	0.14
Anxiety (%)	43	18	<0.01	33	16	<0.01	60	0.03	<0.01
mMRC	3.89 ± 0.32	3.70 ± 0.69	0.25	3.91 ± 0.38	3.65 ± 0.73	<0.01	3.94 ± 0.25	0.40	0.75
CFS	5.0/5.0–6.0/	5.0/4.0–6.0/	0.10	5.0/5.0–6.0/	5.0/4.0–6.0/	0.08	6.0/5.0–6.3/	0.16	0.10
FEV_1_ (% pred)	34/24–42/	39/27–50/	0.10	36/25–48/	39/27–51/	0.39	35/24–45/	0.79	0.45
FVC (% pred)	78/66–82/	69/58–85/	0.54	72/58–85/	69/59–85/	0.84	78/76–82/	0.30	0.28
Pre–NIV setup pH	7.41/7.38–7.44/	7.41/7.35–7.45/	0.56	7.41/7.37–7.44/	7.41/7.35–7.45/	0.93	7.41/7.38–7.43/	0.63	0.79
Pre–NIV setup pO_2_ (kPa)	7.50/6.85–8.30/	7.80/7.20–8.50/	0.33	7.80/7.10–8.50/	7.80/7.20–8.40/	0.99	7.65/7.00–8.53/	0.39	0.66
Pre–NIV setup pCO_2_ (kPa)	7.60/6.85–8.25/	7.80/6.90–8.95/	0.36	7.65/6.98–8.80/	7.75/6.80–8.88/	0.76	7.40/6.88–7.83/	0.80	0.79
ODI (1/h)	6.0/3.7–11.6/	11.5/7.2–27.0/	<0.01	9.6/4.2–21.2/	11.7/6.5–23.7/	0.16	3.8/2.5–6.7/	0.12	0.09
T90% (%)	96.1/72.5–99.7/	89.6/62.0–99.1/	0.39	94.3/69.4–99.2/	89.2/62.3–99.3/	0.58	98.7/95.3–99.9/	0.04	0.09

BMI—body mass index, CCI—Charlson Comorbidity index, CFS—Clinical Frailty Score, FEV_1_—forced expiratory volume in 1 s, FVC—forced vital capacity, mMRC—modified Medical Research Council questionnaire, NIV—non–invasive ventilation, ODI –oxygen desaturation index, T90%—time spent with oxygen saturation below 90%. *p*-value 1—Patients on benzodiazepines vs. Patients not on benzodiazepines. *p*-value 2—Patients on opioids vs. Patients not on opioids. *p*-value 3—Patients on benzodiazepines and on vs. off opioids. *p*-value 4—Patients on opioids and on vs. off benzodiazepines.

## Data Availability

The data presented in this project are available upon reasonable request from the corresponding author.
